# Smartphones and Medical Applications in the Emergency Department Daily Practice 

**Published:** 2017-01-09

**Authors:** Amirhosein Jahanshir, Ehsan Karimialavijeh, Hojjat Sheikh, Motahar Vahedi, Mehdi Momeni

**Affiliations:** Department of Emergency Medicine, Dr. Shariati Hospital, Tehran University of Medical Sciences, Tehran, Iran.

**Keywords:** Smartphone, mobile applications, emergency service, hospital, evidence-based practice

## Abstract

**Introduction:**

Medical applications help physicians to make more rapid and evidence based decisions that may provide better patient care. This study aimed to determine the extent to which smart phones and medical applications are integrated in the emergency department daily practice.

**Method:**

In a cross sectional study, a modified standard questionnaire (Payne et al.) consisting of demographic data and information regarding quality and quantity of smartphone and medical app utilization was sent to emergency-medicine residents and interns twice (two weeks apart), in January 2015. The questionnaire was put online using open access "Web-form Module" and the address of the web page was e-mailed along with a cover letter explaining the survey. Finally, responses were analyzed using descriptive statistics and SPSS 22 software.

**Results:**

65 cases participated (response rate 86%). The mean age of interns and residents were 25.03 ± 1.13 and 30.27 ± 4.68 years, respectively (p < 0.001). There was no significant difference between interns and residents in owning a smartphone (p = 0.5). Android was more popular than IOS (67.7% against 25.8%) and the most popular medical apps were Medscape and UpToDate, respectively. 38 (61.3%) of the respondents were using their apps more than once a day and mostly for drug information. English (83.9%), Persian (12.9%), and other languages (3.2%) were preferred languages for designing a medical software among the participants, respectively.

**Conclusion:**

The findings of present study showed that smartphones are very popular among Iranian interns and residents in emergency department and a substantial number of them own a smartphone and are using medical apps regularly in their clinical practice.

## Introduction

Today smartphones and tablets are universal, well known, and popular devices that are integrated into daily life of many people (1). By installing appropriate mobile applications on a smartphone or tablet, they will be capable of performing different tasks. App is short for application, and a mobile app is a software that has been designed to run on smartphones or tablets. Apps can be downloaded from application distribution platforms (ADP) such as Google Play, App Store, Windows Phone Store, and BlackBerry App World. International sanctions on Iran led to development of local Iranian app stores like Bazaar and Myket, which serve as sources of mobile apps.

For healthcare professionals, this novel technology provides an opportunity to promote patient care and decrease medical errors through rapid access to the latest evidence based medical information (2, 3). Physicians use medical apps for different purposes such as: learning, education, decision making, medical calculation, and better interpretation of paraclinical tests (4-11). Medical students are also very familiar with these handheld devices (12, 13). 

Despite all benefits, several problems exist in this context. Smartphones may have hardware limitations such as narrow screen, connectivity issues and so on. The reliability of medical apps is also under debate (14). The problem of app overload is another factor that may also confuse users in finding appropriate applications (15).

Based on the above mentioned, the present study aimed to investigate the extent of smartphone ownership and utilization of medical applications among interns and residents in an emergency department.

## Methods

***Study design and setting***

This cross sectional study was performed in a teaching hospital of Tehran University of Medical Sciences (TUMS) in Iran, during January 2015. Who met the inclusion criteria were contacted via 2 separate emails (two weeks apart). Inclusion criteria consisted of 1. Participation in TUMS emergency medicine training program; 2. Registration of email address into the database of office of the vice chancellor for student affairs; 3. Consent for use of mail address in correspondence with TUMS. The local ethical committee of TUMS approved the conduct of the study and researchers adhered to all Helsinki recommendations and confidentiality of participants’ information.

***Data gathering***

Initially, a modified version of Payne et al. questionnaire of smartphones and medical apps use among medical students was developed (1). The questionnaire was translated into Persian by the authors separately, and then the best translation for each item was chosen. Statements about preferred apps and their user interface language were added, and one statement about current hospital of employment was deleted. Medical apps were mentioned in the questionnaire, according to the researchers’ personal experience about their possible popularity. In order to establish face validity of the survey, an expert panel of emergency medicine professors, who were familiar with new technologies and medical applications, reviewed the final questionnaire and compared it with the original one. They suggested 2 minor revisions in translation and approved its content validity. 

30 interns, who were in the emergency department rotation, were asked to fill the questionnaire as a pilot study. Principle components analysis (PCA) was performed and the Cronbach's alpha coefficient was calculated to evaluate the internal consistency of the survey. Values higher than 0.6, were considered acceptable. 

The questionnaire was put online using open access "Web-form Module" in a website that was designed with "Drupal Platform", and the address of the web page as well as a cover letter explaining the survey were sent to 75 emergency department residents and interns by email and they were asked to fill it. Responses were IP-sensitive and stored on a password-protected server. If any of the participants wanted to fill the questionnaire in an offline format, printed questionnaires were available.

***Measured items***

1. The number of smartphone owners among emergency medicine residents and interns.

 2. The frequency of smartphone operating systems (IOS, Android, Windows mobile, etc.).

3. The frequency of medical applications among smartphone owners. 

4. The rate of medical app utilization among emergency medicine residents and interns.

***Statistical analysis***

Data analysis was performed using SPSS version 22. Data were analyzed using descriptive statistics. Quantitative variables were reported as mean and standard deviation and qualitative ones as frequency and percentage. The results of the pilot study were not included in the final analysis. Student *t*-test was used for comparing the mean age of interns and residents. P < 0.05 was considered statistically significant.

## Results

The survey of 65 participants (response rate 86%) was analyzed (50.8% female). 62 (95.38%) of them owned a smartphone. Table 1 summarizes the baseline characteristics of participants. The mean age of interns and residents were 25.03 ± 1.13 and 30.27 ± 4.68 years, respectively (p < 0.001). There was no significant difference between interns and residents in owning a smartphone (p = 0.5). Table 2 shows the popularity of smartphone operating systems and medical apps as well as frequency of their daily use and indications. Android was more popular than IOS (67.7% against 25.8%) and the most popular medical apps were Medscape and UpToDate, respectively. 38 (61.3%) of the respondents were using their apps more than once a day and mostly for drug information. 

**Figure 1:**
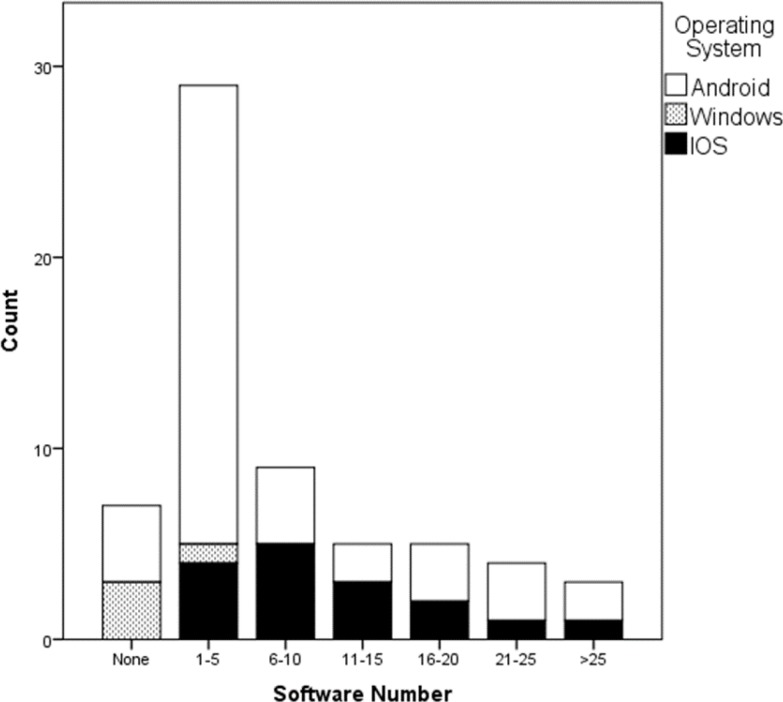
Distribution of installed apps and operating systems among the respondents.

Figure 1 shows the distribution of medical apps installed on different smartphone operating systems (range 0 to 25 apps). Although WikEM was the only emergency medicine app that was mentioned in the questionnaire, 61 (98.4%) participants had not installed it on their smartphones. English (83.9%), Persian (12.9%), and other languages (3.2%) were preferred languages for designing a medical software among the participants, respectively.

**Table 1:** Baseline characteristics of the participants (n=65)

**Items**	**Number (%)**
**Sex**	
Male	32 (49.2)
Female	33 (50.8)
**Level of education**	
Interns	39 (60)
1^th^ year resident	5 (7.7)
2^nd^ year resident	15 (23.1)
3^th ^year resident	6 (9.2)
**Owned a Smartphone**	
Yes	62 (95.4)
No	3 (4.6)

**Table 2:** Popularity of operating systems and medical apps among study participants

**Variable (n = 62)**	**Number (%)**
**Operating systems**	
Android	42 (67.7)
IOS	16 (25.8)
Windows	4 (6.5)
**Frequency of app use**	
Never	2 (3.2)
Rarely	4 (6.5)
Once a week	5 (8.1)
2-3 times a week	13 (21.0)
1-2 times a day	18 (29.0)
Many times a day	20 (32.3)
**Medical apps**	
Medscape	53 (85.5)
Pubmed	7 (11.3)
Omnio	8 (12.9)
WikEM	1 (1.6)
Up To Date	27 (43.5)
Others	12 (19.4)
**Usage indication**	
Drug information	53 (85.5)
Differential Diagnosis	27 (43.5)
Diagnosis	26 (41.9)
Treatment	38 (61.3)
Procedural Skills	16 (25.8)
Others	9 (14.5)

## Discussion

The findings of the present study showed that smartphones are very popular among Iranian interns and residents and a substantial number of them own a smartphone and are using medical apps regularly in their clinical practice. 

It was shown that using handheld computers and smartphones saves time and expedites decision making and treatment in both pre-hospital and hospital settings (2, 3, 16, 17). It also makes patient care more evidence based and prevents medication errors (3). In emergency medicine, there are guidelines and protocols to minimize wasted time in emergency situations, but when considering patient care, it is reasonable to spend a few moments on finding appropriate information. Previous studies claimed that residents and younger physicians are more interested in using smartphones (18). Although interns were significantly younger than residents in this study, no significant difference was found between them in owning smartphones. Although it is easier to read a text in one's native language, English was the most preferred language for designing a medical app among the respondents. 

In this study, most interns and residents were using medical apps for finding drug information and treatment options. Although neither the information they needed to find on their smartphones nor the amount of time they spent on finding needed information were studied, it is thought that checking necessary information in a critical situation, e.g. epinephrine dose in anaphylaxis, might have a negative impact on patient outcome. An impact study is needed in this regard.

No data could be found about market share of operating systems in Iran. In 2014, the International Data Corporation (IDC) stated that Android, IOS, Windows Phone and Blackberry OS have 76.6%, 19.7%, 2.8% and 0.4% of the worldwide smartphone market share, respectively (19). In this study, 25% of participants were using IOS. The market share of operating systems is entirely related to brands and manufacturers. If we assume that the market share of these operating systems in Iran is similar to what was found among the participants, then we can conclude that the market shares are not significantly different from IDC report and international sanctions on Iran could not change the market shares. This is mostly because 85% of smartphones and tablets are imported into Iran illegally (20).

Although respondents stated that Medscape and UpToDate were the most used medical apps in their smartphones, but what they were using as UpToDate was actually an offline version of UpToDate website, which is not really an app. WikEM, which is a free emergency medicine app, was not popular among the respondents. Despite international sanctions on Iran, which has restricted the access to app stores and some medical apps from Iran, smartphones and medical apps are as popular as they are in other countries and Iranian emergency physicians use them regularly in their clinical practice. 

It seems that purchasing a smartphone or installing a medical app, is a function of the physicians' need rather than their age. Although there are evidence that suggest the use of medical apps may improve patient care in the hospitals, this needs to be evaluated in emergency situations. In critical situations such as the emergency department and its acute area, using a medical app to find the best drug or its dose is time consuming and may defeat the purpose of patient safety.

## Limitation:

Only interns and residents were asked to fill out the questionnaire; therefore, the popularity of new technologies among younger people may interfere with the generalizability of the results of this study.

## Conclusion:

The findings of the present study showed that smartphones are very popular among Iranian interns and residents in emergency department and a substantial number of them own a smartphone and are using medical apps regularly in their clinical practice. 
